# Management of venous thrombosis in sickle cell disease: a comparative study on the use of direct oral anticoagulants and warfarin

**DOI:** 10.1016/j.rpth.2026.103397

**Published:** 2026-02-13

**Authors:** Mosaad Almegren, Eysa N. AlSolamy, Rayan A. Qutob, Mohammed M. Alasmari, Ali Aljeraiwi, Noura Aljuwaisri, Turki Abdulaziz Alshuaibi, Faisal Bahammam, Bader Al Rawahi, Hoor Hamed Al Farsi

**Affiliations:** 1 Department of Medicine, College of Medicine, Imam Mohammad Ibn Saud Islamic University, Saudi Arabia; 2 Department of Medicine, King Fahad Armed Forces, Jeddah, Saudi Arabia; 3 Department of Medicine, Farwaniya and Jaber Al-Ahmad Hospital, Farwaniya Area, Kuwait; 4 Department of Medicine, Amiri Hospital, Kuwait City, Kuwait; 5 Department of Medicine, King Fahad General Hospital, Jeddah, Saudi Arabia; 6 Department of Medicine, National Hematology and Bone Marrow Transplant Center, University Medical City, Sultan Qaboos University, Seeb, Oman; 7 Department of Medicine, Sultan Qaboos University Hospital, Muscat, Oman

**Keywords:** bleeding, direct oral anticoagulants, sickle cell disease, venous thromboembolism, warfarin

## Abstract

**Background:**

Sickle cell disease (SCD) is associated with an increased risk of thrombosis and often leads to mortality.

**Objectives:**

This study aimed to compare the clinical outcomes of direct oral anticoagulants (DOACs) with warfarin in the management of patients with SCD and first venous thrombosis.

**Methods:**

This retrospective study included adult patients aged ≥ 18 years with SCD who developed their first episode of venous thrombotic event. Recurrent thrombosis and bleeding were compared between patients treated with DOACs and warfarin. Data were analyzed using IBM Statistical Package for the Social Sciences version 21.

**Results:**

We included 99 patients, of whom 67 (67.7%) were treated with DOACs and 32 (32.3%) with warfarin. The median follow-up time was 44 (1-130) months. Pulmonary embolism was the most common type of thrombosis observed in 64 patients (64.6 %). Three patients developed recurrent venous thromboembolism within 6 months of the first episode, whereas 6 patients developed recurrent thrombosis after 1 year. No significant difference was noted among patients on either type of anticoagulation in terms of major bleeding episodes (OR = 1.1; 95% CI: 1.1-1.8; *P*: 1.00), recurrence of thrombosis (OR = 0.68; 95% CI: 0.03-11.2; *P*: .68), or mortality (OR = 0.46; 95% CI: 0.06-3.4; *P*: .59). Clinically relevant nonmajor bleeding was significantly lower in patients on DOACs than those on warfarin (OR = 0.06; 95% CI: 0.01-0.52; *P*: .01).

**Conclusion:**

DOACs are associated with similar clinical outcomes and fewer bleeding complications as compared to warfarin in the management of patients with SCD and thrombosis. Randomized controlled trials are required to further confirm our findings.

## Essentials

• Data on the use of direct oral anticoagulants (DOACs) in patients with sickle cell disease and venous thrombosis are limited.
• We aimed to compare DOACs and warfarin in sickle cell disease patients with venous thrombosis.
• No significant difference between DOACs and warfarin in terms of recurrent thrombosis and major bleeding.
• Clinically relevant nonmajor bleeding is significantly lower with the use of DOACs.

## 1 Introduction

Sickle cell disease (SCD) is a global public health concern. The prevalence of SCD varies worldwide, with the highest disease burden observed in Sub-Saharan Africa [1]. In Saudi Arabia, the carrier rate of sickle cell trait varies from 2% to 27%, with the highest rates of SCD observed in the Eastern and Southern provinces [2,3]. The high prevalence of SCD can be attributed to the high rate of consanguineous marriages in Saudi Arabia. The Saudi Ministry of Health has implemented premarital screening programs to mitigate the impact of SCD and other genetic disorders at the national level [4].

SCD is often complicated by a vaso-occlusive crisis, a state in which sickle-shaped red blood cells obstruct blood flow, leading to end-organ damage [5]. Owing to the turbidity of blood flow, vaso-occlusive crisis are associated with a hypercoagulable state, which predisposes patients to the development of blood clots [6]. Furthermore, increased levels of circulating tissue factors, increased platelet activation and thrombin generation, and decreased levels of natural anticoagulants in SCD create a high risk of thrombosis [7]. These factors are further exacerbated by the use of central venous catheters in patients who require frequent blood transfusions [8]. An estimated 11% of SCD patients experience a venous thromboembolism (VTE) event before they reach 40 years of age, with a 5-year recurrence rate reaching up to 36% [9,10]. Thrombosis is a significant cause of morbidity and mortality in patients with SCD [11].

Oral anticoagulants, such as vitamin K antagonists (VKAs) remained the mainstay for management of coagulopathies for a very long time in patients with a confirmed diagnosis of VTE [12]. Warfarin is the oldest and most reported VKA used as part of anticoagulation therapy in high-risk patients. However, the use of VKAs has also been linked to some drawbacks, such as the risk of drug interactions with several medications, a narrow therapeutic window that requires frequent monitoring of the international normalized ratio, and even food interactions with foods rich in vitamin K [13]. Direct oral anticoagulants (DOACs) such as rivaroxaban, dabigatran, edoxaban, and apixaban have helped overcome these limitations and offer several advantages, including a reduced risk of hemorrhagic complications and a reduced requirement for continuous laboratory monitoring, thus improving patient adherence [14]. This study aimed to compare the use of DOACs with that of warfarin in the management of patients with SCD who developed venous thrombosis.

## 2 Methods

This multicenter retrospective study was conducted at 3 tertiary care hospitals in Saudi Arabia, Oman, and Kuwait. All adult patients aged ≥ 18 years who developed first venous thrombosis with an underlying SCD diagnosis were included. Patients who presented to hospitals from January 2013 to January 2023 were included in this study. We excluded carriers of sickle cell trait, pregnant women, posttransplant patients, patients on anticoagulation for reasons other than VTE, and patients with underlying thrombophilia.

### 2.1 Data extraction

Standardized data collection forms were used to record relevant patient demographics and outcomes of interest. Data related to patient demographics, types of SCD, types of VTE, type and time of anticoagulation, laboratory data, medication history, VTE recurrence, bleeding episodes, and mortality were extracted from medical records. Following the diagnosis of VTE, the patients were categorized according to the anticoagulation class. In the case of a switch from one class of anticoagulant to another, patients were classified into the category of anticoagulation they were on at the time of the outcome. The baseline VTE-BLEED score (active cancer, male with uncontrolled arterial hypertension, anemia, history of bleeding, age≥60 years old, renal dysfunction) which estimates the risk of bleeding while on anticoagulation therapy by computing a score based on age, underlying hypertension, malignancy, renal function levels, anemia, and history of bleeding, was determined for each patient [15].

### 2.2 Study outcomes

This study aimed to assess the clinical outcomes of DOAC in patients with SCD and first venous thrombosis compared with those of warfarin. Newly diagnosed recurrent VTE was reported within 6 and 12 months of anticoagulation therapy initiation or during the study period, and any bleeding events that occurred after initiation of anticoagulant therapy were recorded. The bleeding episodes were further classified into major and nonmajor categories. Major bleeding was defined according to the criteria published by the International Society on Thrombosis and Hemostasis (ISTH), which includes bleeds that lead to a drop in hemoglobin of at least 2 g/dL, requiring transfusion of 2 or more units of blood, bleeding that occurs in critical organs, or results in mortality [16].

### 2.3 Statistical analysis

Descriptive statistics were used to define patient demographics in terms of frequencies, mean ± standard deviation (SD), and percentages. The strength of the association between drug class, VTE, and bleeding events was calculated as odds ratios (ORs) with respect to the class of anticoagulant used. Chi-squared tests, Fisher’s exact tests, and independent sample *t*-tests were used to determine the differences between the 2 groups, as appropriate. The statistical significance threshold was defined as *P* value of <.05. Statistical analyses were performed using the Statistical Package for the Social Sciences version 21 (IBM).

### 2.4 Ethical considerations

This study was conducted after receiving ethical approval from the institutional review boards of the participating hospitals. The authors declare no conflict of interest.

## 3 Results

This study included 99 patients with SCD and venous thrombosis, of whom 67 (67.7%) were on DOACs, and 32 (32.3%) received warfarin therapy. The DOACs used were rivaroxaban in 44 patients, apixaban in 21 and dabigatran in 2 patients. The mean age of all study participants was 32.8 ± 9.9 years and majority (60.6%) were women. Sixty-three patients (63.6 %) were homozygous for hemoglobin S, the mutated form of hemoglobin responsible for SCD. The remaining 36 (36.4%) were compound heterozygotes for sickle cell syndromes, including sickle-β thalassemia and other variant hemoglobins. Upon presentation to the hospital, the mean hemoglobin level was 8.7 ± 2.0 g/dL. The frequency of acute chest syndrome was higher in patients treated with DOACs than in those treated with warfarin (*P* = .02). Baseline risk of bleeding was similar for the 2 groups of patients receiving DOACs or warfarin. Table 1 summarizes the demographic and baseline characteristics of the study population.

**Table 1:** Patient demographics and baseline hematological characteristics.

Characteristics	Total (*N* = 99)	DOACs (*n* = 67)	Warfarin (*n* = 32)	*P*
Mean age ± SD	32.8 ± 9.9	32.9 ± 9.9	32.8 ± 10.2	.71
Males, *n* (%)	39 (39.4)	26 (38.9)	13 (40.6)	.86
Type of sickle cell disease, *n* (%)				.82
SS	63 (63.6)	42 (62.7)	21 (65.6)	
Sß+	19 (19.2)	13 (19.4)	6 (18.7)	
Sß0	7 (7.1)	6 (8.9)	1 (3.1)	
SC	1 (1)	1 (1.5)	0 (0)	
Others	8 (8.1)	5 (7.5)	3 (9.4)	
Co-morbidities, *n* (%)				
Bleeding history	9 (9.1)	7 (10.5)	2 (6.3)	.72
Vaso-occlusive crisis	65 (65.7)	47 (70.1)	18 (56.3)	.17
Cardiomyopathy	5 (5.1)	3 (4.5)	2 (6.3)	.66
Ischemic stroke	10 (10.4)	8 (11.9)	2 (6.3)	.49
Acute chest syndrome	41 (41.4)	33 (49.2)	8 (25.0)	**.02**
Hyperhemolytic crisis	4 (4)	1 (1.5)	3 (9.4)	.098
Avascular necrosis	31 (31.3)	23 (34.3)	8 (25.0)	.35
Diabetes	1 (1)	0 (0)	1 (3.1)	.32
Hypertension	8 (8.1)	4 (6.0)	4 (12.5)	.27
Platelet count (x 10^3^/uL)	381.3 ± 242.8	391± 246.3	359.5 ± 237.9	.56
Hemoglobin (g/dL)	8.7 ± 2.0	8.9 ± 1.6	8.3 ± 2.7	.51
VTE-BLEED score, Mean ± SD	2.0 ± 0.97	2.1 ± 0.87	2.0 ± 1.2	.57

1 significant *P* value is indicated in bold.

DOAC, direct oral anticoagulants.

Pulmonary embolism (PE) was the most common type of thrombosis, encountered in 64 patients (64.6 %). This was followed by proximal deep vein thrombosis (DVT) in 23 (23.2%), catheter-related DVT in 5 (5.1%), and distal DVT in 3 (3%) patients. Other rare thrombotic events included cerebral vein thrombosis in 3 (3%), superior vena cava thrombosis in 2, portal vein thrombosis in one, and Budd-Chiari syndrome in one. Two patients with superior vena cava thrombosis also had PE, leading to 101 thrombotic events in the 99 patients. The Figure depicts the types of thrombosis and the anticoagulation therapy administered for each type. The median follow-up period for the entire study population was 44 months (range: 1-130 months), while the median duration of anticoagulation was 12.5 months (range: 3-127 months). Sixty-five (64.4%) patients received parenteral anticoagulation therapy before switching to oral anticoagulation therapy. A total of 57 (57.6%) patients also received hydroxyurea at the time of VTE diagnosis.

**Figure:**
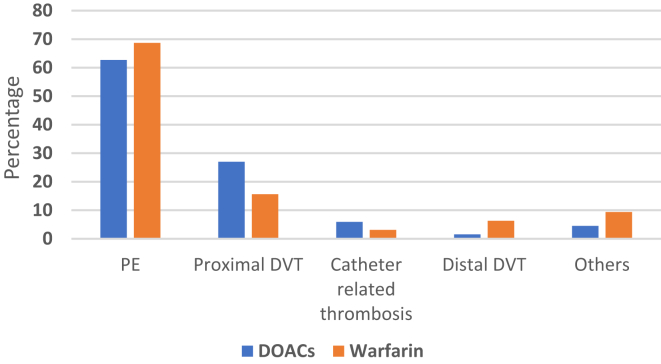
Type of thrombosis and anticoagulation received. DOACs, direct oral anticoagulants; DVT, deep vein thrombosis.

### 3.1 Recurrence of thrombosis

Overall, 9 patients (9.1%) developed venous thrombosis recurrence during the study period. The initial type of thrombosis was PE in 7 (7.1%) patients, proximal DVT in one patient, and catheter-related DVT in one patient. The recurrent thrombotic episodes were DVT in 3 (3%) patients and PE in 6 (6.1) patients.

Among these 9 patients, 3 (3%) had a recurrent thrombotic event within 6 months of the first episode, and 6 (6%) developed recurrent thrombosis >12 months of the first event. No recurrent thrombotic events were noted between 6 and 12 months after VTE occurrence. Four of the patients with recurrent VTE were on hydroxyurea. Among the 3 patients who developed a second thrombosis within 6 months, 2 patients were on a DOAC (rivaroxaban) while one patient was taking warfarin at the time of VTE recurrence. Among the 6 patients who developed late recurrence after 1 year, 2 patients discontinued anticoagulation therapy at the time of VTE, 2 were on DOACs, and 2 were taking warfarin. Fisher’s exact test revealed no significant difference in thrombosis recurrence between patients taking warfarin or DOACs (*P* = .71).

### 3.2 Bleeding complications

Two patients taking DOACs (one on rivaroxaban and one on apixaban) developed major bleeding, which was not observed in any patient taking warfarin. Among the patients with minor or clinically relevant nonmajor bleeding, 4 patients were on warfarin, while 3 were on DOACs (rivaroxaban). The risk of clinically relevant nonmajor bleeding was significantly lower in patients receiving DOACs (*P* = .012). All bleeding episodes occurred during out-patient follow-up.

Overall, 4 patients died during the study period. Table 2 summarizes the outcomes of the study population.

**Table 2:** Outcomes of our study population categorized according to the type of anticoagulation received.

Outcome	DOACs (*n* = 67) *n* (%)	Warfarin (*n* = 32) *n* (%)	Risk OR (95% CI)	*P*
Recurrent VTE	4 (6.0)	3 (9.4)	0.68 (0.03-11.29)	.69
Major bleeding	2 (3.0)	0 (0)	1.15 (1.23-1.76)	1.000
Clinically relevant nonmajor bleeding	2 (3.0)	4 (12.5)	0.06 (0.01-0.52)	**.01**
Any bleeding	5 (7.5)	4 (12.5)	0.357 (0.08 - 1.58)	.22
Minor bleeding	1 (1.5)	0 (0)	1.21 (1.02-1.44)	1.000
Death	2 (3.0)	2 (6.3)	0.46 (0.06-3.42)	.59

1 significant *P* value is indicated in bold.

DOAC, direct oral anticoagulants; VTE, venous thromboembolism.

## 4 Discussion

VTE is linked to an increased risk of mortality in patients [17]. Previously, VKAs were used as frontline therapy for the management of thrombosis in SCD. However, in recent years, the advent of DOACs has revolutionized VTE [18]. This study is the first to compare the use of DOACs and warfarin in patients with SCD complicated by thrombosis in the Arabic Gulf region.

The risk of developing clinically relevant nonmajor bleeding during anticoagulation therapy was significantly lower among patients on DOACs than that among those on warfarin. These hemorrhagic complications developed more frequently in patients taking warfarin, although the baseline bleeding risk for patients in both treatment groups was similar. Our finding is similar to that of a study by Patel et al. [19], who found that among 109 patients with SCD and VTE, the incidence of bleeding was significantly higher among patients on VKAs than among those on DOACs (incidence: 6.7 vs 2.5 events per 100 person-years, *P* < .05).

Nine percent of our patients with SCD experienced thrombosis recurrence. A previous study by Brunson et al. [20], which included 877 patients with SCD and VTE, reported that the cumulative incidence of VTE recurrence was 13.2% at 1 year follow-up and 24.1% at 5 years of follow-up. The authors suggested that a high risk of recurrent thrombosis in SCD may require a prolonged duration of thromboprophylaxis; however, the associated risk of bleeding must also be considered in each individual case.

The risk of recurrent thrombosis among our patients was similar to that among patients treated with DOACs and warfarin. Similarly, Roberts et al. [21] reported that among patients with SCD and VTE, 9 (24%) of 37 patients developed recurrent thrombosis. This included 6 (27%) of 22 patients on DOACs compared with 3 (20%) of 15 patients on warfarin. However, another study by Gupta et al. [22] found a much higher rate of VTE recurrence in 27 (49.1%) of the 55 patients with SCD and venous thrombosis. Among them, 4 (27%) patients were on rivaroxaban and 7 (19%) were on warfarin. However, as the overall recurrence rate was much higher than that in previous studies, the authors acknowledged that a prospective study design should be employed to account for confounding factors such as patient adherence to treatment.

No significant differences were observed in the incidence of mortality between patients with SCD treated with DOACs or warfarin. Previous studies have shown comparable survival outcomes in patients with VTE treated with warfarin or DOACs [23]. However, data on survival outcomes in the context of SCD and the choice of anticoagulants are limited.

A systematic review and meta-analysis conducted by Rozi et al. [24], which included 5 studies from the United States and one study from France, reported that among SCD patients with VTE, management with DOACs resulted in similar rates of VTE recurrence and lower rates of bleeding as compared with VKAs and low molecular weight heparin. These findings are similar to those of this study.

This study verified the clinical outcomes of DOACs compared with warfarin in patients with SCD and venous thrombosis. Currently, well-defined thromboprophylaxis guidelines for patients with SCD or VTE are lacking. Therefore, our findings and those of similar reports should be considered when developing population-specific thromboprophylaxis guidelines for this unique group of patients. The strength of our study lies in its robust sample size compared with that of previous studies, which mostly comprised case series with a small number of patients. However, it should be noted that as the number of patients with complications of VTE recurrence or bleeding was small, this limits the statistical power of the risk estimates. Our results should be confirmed through prospective studies with a longer follow-up duration, as well as randomized controlled trials, to eliminate the effect of confounding factors that may have an impact on patient outcomes during anticoagulation therapy.

## 5 Conclusion

This study indicates that DOACs are associated with similar clinical outcomes, comparable recurrence rates, and a relatively lower risk of bleeding than warfarin in the management of patients with SCD with venous thrombosis. However, randomized controlled trials comparing the safety and efficacy of DOACs and VKAs should be conducted for further confirmation.
